# (*S*)-(−)-1-Phenyl­ethanaminium hexa­noate

**DOI:** 10.1107/S1600536812045746

**Published:** 2012-11-10

**Authors:** Mary H. Wood, Stuart M. Clarke

**Affiliations:** a BP Institute and Department of Chemistry, University of Cambridge, Cambridge CB3 0EZ, England

## Abstract

A binary mixture of (*S*)-(−)-1-phenyl­ethanamine and hexa­noic acid was allowed to react to form the title salt, C_8_H_12_N^+^·C_6_H_11_O_2_^−^. This crystal contains a 1:1 stoichiometric mixture of the acid- and amine-derived species and displays a chiral structure with N—H⋯O hydrogen-bonded chains propagating along the *c*-axis direction.

## Related literature

For spectroscopic studies of acid–amine complexes, see: Karlsson *et al.* (2000 ▶); Paivarinta *et al.* (2000 ▶); Kohler *et al.* (1981 ▶); Smith *et al.* (2001 ▶, 2002 ▶); Klokkenburg *et al.* (2007 ▶). For recent diffraction studies of acid–amine complexes, see: Jefferson *et al.* (2011 ▶); Sun *et al.* (2011 ▶); Wood & Clarke (2012 ▶).
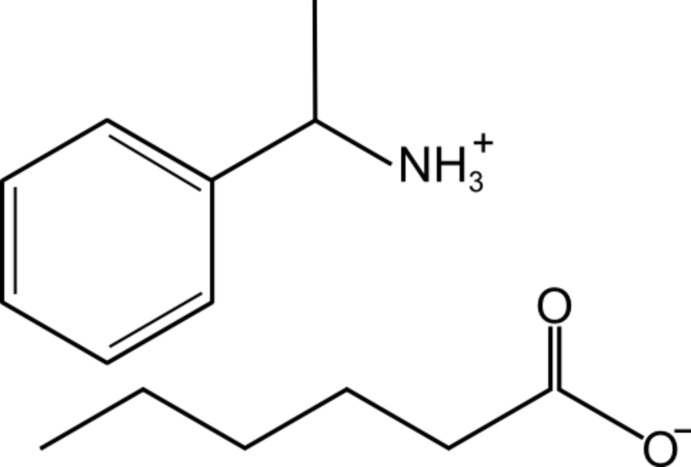


## Experimental

### 

#### Crystal data

C_8_H_12_N^+^·C_6_H_11_O_2_^−^
*M* *_r_* = 237.33
Hexagonal, 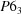

*a* = 19.5845 (5) Å
*c* = 6.6307 (2) Å
*V* = 2202.49 (10) Å^3^
*Z* = 6
Mo *K*α radiation
μ = 0.07 mm^−1^
*T* = 180 K
0.46 × 0.05 × 0.05 mm

#### Data collection

Nonius KappaCCD diffractometer
Absorption correction: multi-scan (*SORTAV*; Blessing, 1995 ▶) *T*_min_ = 0.740, *T*_max_ = 0.999
11638 measured reflections
1461 independent reflections
1270 reflections with *I* > 2σ(*I*)
*R*_int_ = 0.064

#### Refinement

*R*[*F* ^2^ > 2σ(*F* ^2^)] = 0.040
*wR*(*F* ^2^) = 0.087
*S* = 1.06
1461 reflections
157 parameters
1 restraint
H-atom parameters constrained
Δρ_max_ = 0.11 e Å^−3^
Δρ_min_ = −0.14 e Å^−3^

### 

Data collection: *COLLECT* (Nonius, 1998 ▶); cell refinement: *SCALEPACK* (Otwinowski & Minor, 1997 ▶); data reduction: *DENZO* (Otwinowski & Minor, 1997 ▶) and *SCALEPACK*; program(s) used to solve structure: *SIR92* (Altomare *et al.*, 1994 ▶); program(s) used to refine structure: *SHELXL97* (Sheldrick 2008 ▶); molecular graphics: *Mercury* (Macrae *et al.*, 2008 ▶); software used to prepare material for publication: *SHELXL97*.

## Supplementary Material

Crystal structure: contains datablock(s) I, global. DOI: 10.1107/S1600536812045746/mw2095sup1.cif

Structure factors: contains datablock(s) I. DOI: 10.1107/S1600536812045746/mw2095Isup2.hkl

Supplementary material file. DOI: 10.1107/S1600536812045746/mw2095Isup3.cml

Additional supplementary materials:  crystallographic information; 3D view; checkCIF report

## Figures and Tables

**Table 1:** Hydrogen-bond geometry (Å, °)

*D*—H⋯*A*	*D*—H	H⋯*A*	*D*⋯*A*	*D*—H⋯*A*
N1—H1*A*⋯O2^i^	0.91	1.84	2.753 (3)	176
N1—H1*B*⋯O1	0.91	1.87	2.768 (3)	167
N1—H1*C*⋯O1^ii^	0.91	1.82	2.714 (2)	168

Symmetry codes: (i) 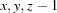; (ii) 
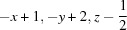
.

**Fig. 1.**
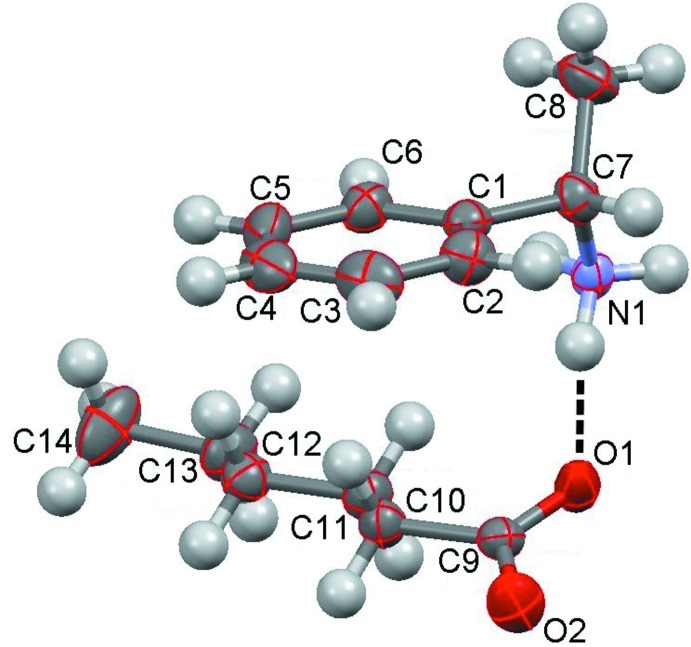
Perspective view of the asymmetric unit showing one of the three N—H···O hydrogen bonds.

**Fig. 2.**
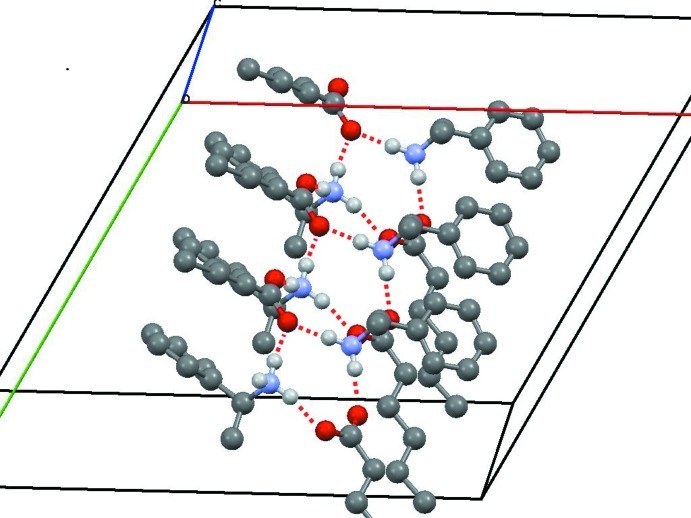
Illustration of the molecular packing - top view of a hydrogen bonded chain. Hydrogen bonds are shown by dashed red lines.

**Fig. 3.**
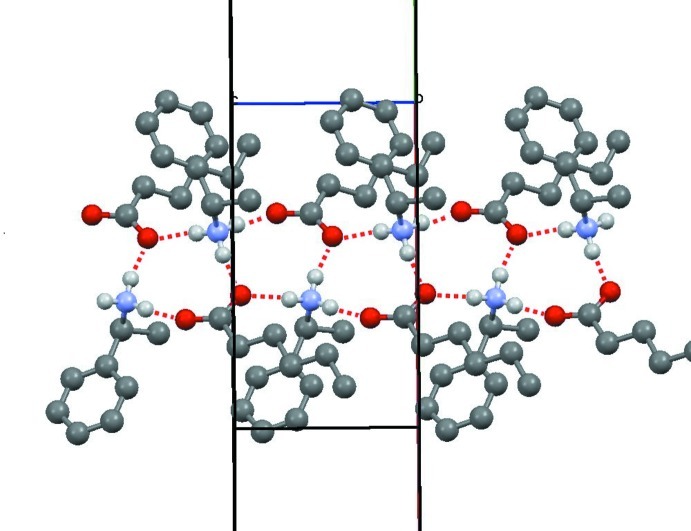
Illustration of the molecular packing - side view of a hydrogen bonded chain. Hydrogen bonds are shown by dashed red lines and form chains of molecules parallel to the c axis.

## supplementary crystallographic information

### Comment

The existence of stable acid:amine complexes formed from simple acid and amine reagents has been reported in the literature (Klokkenburg *et al.*, 2007; Karlsson *et al.*, 2000). Many examples adopt a 1:1 stoichiometry, although acid-rich complexes are not uncommon, with both 2:1 and 3:1 adducts observed in some cases (Sun *et al.*, 2011; Kohler *et al.*, 1981). Amine-rich complexes are thought to be inherently instable and thus unlikely to form (Paivarinta *et al.*, 2000), although there is a report of a diamine complex formed between methylamine and dnsa (3,5-dinitrosalicyclic acid) due to deprotonation of the phenolic group in the acid (Smith *et al.*, 2001; Smith *et al.*, 2002).

The stability of complexes such as the title compound derives from the reactive exchange of a proton giving cations and anions with a strong electrostatic attraction. These ions subsequently interact via strong hydrogen-bond formation; each ammonium ion in the s-(-)-α-methylbenzylammonium hexanoate example is able to form three hydrogen bonds (shown in Figures 1, 2 and 3). For the acid-rich complexes, the hydrogen bonding is considered to extend over the three (or more) species involved.

This work follows previous findings of the formation of a 1:1 complex of octanoic acid and decylamine using the same method (Jefferson *et al.*, 2011) as well as a 1:1 complex between benzylamine and hexanoic acid (Wood *et al.*, 2012). This work focuses on the use of a chiral amine, s-(-)-α-methylbenzylamine.

Whilst spectroscopic studies identifying such acid:amine complexes are reasonably common, there still only a few examples of single-crystal X-ray data, as reported here. This may be attributed to the difficulty of growing suitable crystals as outlined in the experimental section.

### Experimental

Hexanoic acid and s-(-)-methylbenzylamine, with purities of 99.5% and 99.8% respectively as determined by titration and GC, were purchased from Sigma Aldrich and used without further purification. The crystals were grown by pipetting a small volume (approximately 1 ml) of each into small vials and leaving within a larger vial along with a polypropylene nucleation surface under an inert atmosphere (to minimize amine reaction with atmospheric CO_2_ (Sun *et al.*, 2011)). After several weeks abundant crystal growth on the polypropylene surface was observed and a sample selected for X-ray characterization.

Elemental analysis of the crystalline sample gave values of 70.64%, 5.98%, 9.72% and 13.66% for carbon, nitrogen, hydrogen and oxygen respectively. For a 1:1 acid: amine complex, the expected values are: 70.85%, 5.90%, 9.77% and 13.48%, in excellent agreement.

The experimental sample temperature 180 K represents a compromise of improved thermal factors but avoiding sample fracture.

### Refinement

The absolute structure was assigned from the known configuration of the starting material. 1183 Friedel pairs were averaged for the refinement.

Hydrogen site location were inferred from neighbouring sites and H-atom parameters were constrained in the refinement.

### Crystal data

**Table:**

C_8_H_12_N^+^·C_6_H_11_O_2_^−^	*D*_x_ = 1.074 Mg m^−^^3^
*M**_r_* = 237.33	Mo *K*α radiation, λ = 0.71073 Å
Hexagonal, *P*6_3_	Cell parameters from 7677 reflections
Hall symbol: P 6c	θ = 1.0–25.4°
*a* = 19.5845 (5) Å	µ = 0.07 mm^−^^1^
*c* = 6.6307 (2) Å	*T* = 180 K
*V* = 2202.49 (10) Å^3^	Needle, colourless
*Z* = 6	0.46 × 0.05 × 0.05 mm
*F*(000) = 780	

### Data collection

**Table:**

Nonius KappaCCD diffractometer	1270 reflections with *I* > 2σ(*I*)
Radiation source: fine-focus sealed tube	*R*_int_ = 0.064
Thin slice ω and φ scans	θ_max_ = 25.4°, θ_min_ = 3.6°
Absorption correction: multi-scan (*SORTAV*; Blessing, 1995)	*h* = −23→22
*T*_min_ = 0.740, *T*_max_ = 0.999	*k* = −22→23
11638 measured reflections	*l* = −7→7
1461 independent reflections	

### Refinement

**Table:**

Refinement on *F*^2^	Primary atom site location: structure-invariant direct methods
Least-squares matrix: full	Secondary atom site location: difference Fourier map
*R*[*F*^2^ > 2σ(*F*^2^)] = 0.040	Hydrogen site location: inferred from neighbouring sites
*wR*(*F*^2^) = 0.087	H-atom parameters constrained
*S* = 1.06	*w* = 1/[σ^2^(*F*_o_^2^) + (0.0379*P*)^2^ + 0.3614*P*] where *P* = (*F*_o_^2^ + 2*F*_c_^2^)/3
1461 reflections	(Δ/σ)_max_ < 0.001
157 parameters	Δρ_max_ = 0.11 e Å^−^^3^
1 restraint	Δρ_min_ = −0.14 e Å^−^^3^

### Special details

**Table:**

Experimental. multi-scan from symmetry-related measurements Sortav (Blessing, 1995)
Geometry. All e.s.d.'s (except the e.s.d. in the dihedral angle between two l.s. planes) are estimated using the full covariance matrix. The cell e.s.d.'s are taken into account individually in the estimation of e.s.d.'s in distances, angles and torsion angles; correlations between e.s.d.'s in cell parameters are only used when they are defined by crystal symmetry. An approximate (isotropic) treatment of cell e.s.d.'s is used for estimating e.s.d.'s involving l.s. planes.
Refinement. Refinement of *F*^2^ against ALL reflections. The weighted *R*-factor *wR* and goodness of fit *S* are based on *F*^2^, conventional *R*-factors *R* are based on *F*, with *F* set to zero for negative *F*^2^. The threshold expression of *F*^2^ > σ(*F*^2^) is used only for calculating *R*-factors(gt) *etc*. and is not relevant to the choice of reflections for refinement. *R*-factors based on *F*^2^ are statistically about twice as large as those based on *F*, and *R*- factors based on ALL data will be even larger.

### Fractional atomic coordinates and isotropic or equivalent isotropic displacement parameters (Å^2^)

**Table:**

	*x*	*y*	*z*	*U*_iso_*/*U*_eq_	
N1	0.51079 (11)	0.94274 (11)	0.0797 (3)	0.0332 (4)	
H1A	0.5437	0.9491	−0.0246	0.050*	
H1B	0.5394	0.9618	0.1949	0.050*	
H1C	0.4849	0.9695	0.0530	0.050*	
C1	0.49217 (13)	0.81354 (13)	0.1884 (4)	0.0352 (5)	
C2	0.46986 (15)	0.77731 (15)	0.3746 (4)	0.0442 (6)	
H2	0.4295	0.7797	0.4483	0.053*	
C3	0.50523 (17)	0.73764 (15)	0.4557 (5)	0.0530 (7)	
H3	0.4899	0.7139	0.5850	0.064*	
C4	0.56286 (17)	0.73265 (15)	0.3483 (5)	0.0534 (8)	
H4	0.5871	0.7051	0.4027	0.064*	
C5	0.58501 (16)	0.76781 (15)	0.1621 (5)	0.0497 (7)	
H5	0.6246	0.7642	0.0879	0.060*	
C6	0.55026 (14)	0.80857 (15)	0.0807 (4)	0.0415 (6)	
H6	0.5662	0.8329	−0.0479	0.050*	
C7	0.45226 (13)	0.85703 (14)	0.1056 (4)	0.0367 (6)	
H7	0.4121	0.8522	0.2063	0.044*	
C8	0.41036 (17)	0.82438 (18)	−0.0939 (5)	0.0580 (8)	
H8A	0.3859	0.8549	−0.1387	0.087*	
H8B	0.3696	0.7690	−0.0765	0.087*	
H8C	0.4486	0.8281	−0.1951	0.087*	
O1	0.57647 (10)	0.99218 (11)	0.4586 (3)	0.0445 (5)	
O2	0.60989 (10)	0.96859 (11)	0.7585 (3)	0.0492 (5)	
C9	0.61695 (13)	0.97445 (13)	0.5718 (4)	0.0326 (5)	
C10	0.67789 (14)	0.95875 (14)	0.4762 (4)	0.0360 (5)	
H10A	0.6532	0.9013	0.4529	0.043*	
H10B	0.7211	0.9736	0.5745	0.043*	
C11	0.71404 (14)	1.00073 (15)	0.2782 (4)	0.0396 (6)	
H11A	0.6714	0.9874	0.1792	0.047*	
H11B	0.7414	1.0584	0.3008	0.047*	
C12	0.77217 (14)	0.97868 (14)	0.1911 (4)	0.0396 (6)	
H12A	0.8136	0.9902	0.2926	0.048*	
H12B	0.7442	0.9212	0.1647	0.048*	
C13	0.81090 (17)	1.02175 (16)	−0.0018 (4)	0.0495 (7)	
H13A	0.8426	1.0789	0.0267	0.059*	
H13B	0.7695	1.0138	−0.1000	0.059*	
C14	0.8639 (2)	0.99434 (19)	−0.0959 (5)	0.0714 (10)	
H14A	0.8851	1.0221	−0.2237	0.107*	
H14B	0.8333	0.9374	−0.1208	0.107*	
H14C	0.9075	1.0058	−0.0037	0.107*	

### Atomic displacement parameters (Å^2^)

**Table:**

	*U*^11^	*U*^22^	*U*^33^	*U*^12^	*U*^13^	*U*^23^
N1	0.0388 (10)	0.0438 (11)	0.0260 (9)	0.0273 (9)	0.0000 (9)	−0.0005 (9)
C1	0.0343 (12)	0.0335 (12)	0.0342 (13)	0.0143 (10)	−0.0028 (11)	−0.0051 (12)
C2	0.0440 (14)	0.0454 (14)	0.0401 (15)	0.0202 (12)	0.0053 (12)	0.0048 (12)
C3	0.0592 (17)	0.0431 (15)	0.0479 (18)	0.0189 (14)	−0.0035 (15)	0.0121 (14)
C4	0.0577 (18)	0.0333 (14)	0.070 (2)	0.0229 (13)	−0.0176 (16)	−0.0024 (15)
C5	0.0481 (15)	0.0459 (15)	0.0634 (19)	0.0297 (13)	−0.0045 (15)	−0.0113 (15)
C6	0.0441 (14)	0.0422 (14)	0.0392 (13)	0.0223 (12)	0.0030 (13)	−0.0027 (12)
C7	0.0317 (12)	0.0433 (13)	0.0362 (14)	0.0195 (11)	0.0033 (11)	0.0028 (11)
C8	0.0509 (17)	0.0625 (18)	0.059 (2)	0.0272 (14)	−0.0232 (15)	−0.0104 (16)
O1	0.0534 (10)	0.0647 (11)	0.0333 (10)	0.0430 (9)	−0.0058 (9)	−0.0097 (9)
O2	0.0512 (11)	0.0703 (13)	0.0298 (11)	0.0331 (10)	0.0079 (9)	0.0073 (9)
C9	0.0327 (12)	0.0322 (12)	0.0300 (15)	0.0142 (10)	−0.0018 (11)	−0.0032 (11)
C10	0.0373 (13)	0.0420 (13)	0.0321 (13)	0.0223 (11)	−0.0006 (11)	−0.0004 (11)
C11	0.0427 (14)	0.0453 (14)	0.0339 (14)	0.0244 (12)	0.0017 (11)	0.0003 (12)
C12	0.0361 (13)	0.0390 (13)	0.0406 (14)	0.0164 (11)	0.0013 (12)	−0.0020 (12)
C13	0.0524 (16)	0.0557 (16)	0.0407 (14)	0.0272 (14)	0.0092 (13)	0.0006 (14)
C14	0.084 (2)	0.0603 (19)	0.072 (2)	0.0374 (17)	0.0398 (19)	0.0078 (17)

### Geometric parameters (Å, º)

**Table:**

N1—C7	1.496 (3)	C8—H8C	0.9800
N1—H1A	0.9100	O1—C9	1.260 (3)
N1—H1B	0.9100	O2—C9	1.244 (3)
N1—H1C	0.9100	C9—C10	1.513 (3)
C1—C2	1.382 (4)	C10—C11	1.522 (4)
C1—C6	1.387 (3)	C10—H10A	0.9900
C1—C7	1.518 (3)	C10—H10B	0.9900
C2—C3	1.382 (4)	C11—C12	1.519 (3)
C2—H2	0.9500	C11—H11A	0.9900
C3—C4	1.378 (4)	C11—H11B	0.9900
C3—H3	0.9500	C12—C13	1.511 (4)
C4—C5	1.374 (4)	C12—H12A	0.9900
C4—H4	0.9500	C12—H12B	0.9900
C5—C6	1.391 (4)	C13—C14	1.520 (4)
C5—H5	0.9500	C13—H13A	0.9900
C6—H6	0.9500	C13—H13B	0.9900
C7—C8	1.519 (4)	C14—H14A	0.9800
C7—H7	1.0000	C14—H14B	0.9800
C8—H8A	0.9800	C14—H14C	0.9800
C8—H8B	0.9800		
			
C7—N1—H1A	109.5	H8B—C8—H8C	109.5
C7—N1—H1B	109.5	O2—C9—O1	124.2 (2)
H1A—N1—H1B	109.5	O2—C9—C10	117.4 (2)
C7—N1—H1C	109.5	O1—C9—C10	118.4 (2)
H1A—N1—H1C	109.5	C9—C10—C11	116.9 (2)
H1B—N1—H1C	109.5	C9—C10—H10A	108.1
C2—C1—C6	118.9 (2)	C11—C10—H10A	108.1
C2—C1—C7	119.5 (2)	C9—C10—H10B	108.1
C6—C1—C7	121.6 (2)	C11—C10—H10B	108.1
C1—C2—C3	121.2 (3)	H10A—C10—H10B	107.3
C1—C2—H2	119.4	C12—C11—C10	112.7 (2)
C3—C2—H2	119.4	C12—C11—H11A	109.0
C4—C3—C2	119.8 (3)	C10—C11—H11A	109.0
C4—C3—H3	120.1	C12—C11—H11B	109.0
C2—C3—H3	120.1	C10—C11—H11B	109.0
C5—C4—C3	119.5 (3)	H11A—C11—H11B	107.8
C5—C4—H4	120.2	C13—C12—C11	113.7 (2)
C3—C4—H4	120.2	C13—C12—H12A	108.8
C4—C5—C6	120.9 (3)	C11—C12—H12A	108.8
C4—C5—H5	119.5	C13—C12—H12B	108.8
C6—C5—H5	119.5	C11—C12—H12B	108.8
C1—C6—C5	119.6 (3)	H12A—C12—H12B	107.7
C1—C6—H6	120.2	C12—C13—C14	113.0 (2)
C5—C6—H6	120.2	C12—C13—H13A	109.0
N1—C7—C1	110.52 (17)	C14—C13—H13A	109.0
N1—C7—C8	108.8 (2)	C12—C13—H13B	109.0
C1—C7—C8	113.6 (2)	C14—C13—H13B	109.0
N1—C7—H7	107.9	H13A—C13—H13B	107.8
C1—C7—H7	107.9	C13—C14—H14A	109.5
C8—C7—H7	107.9	C13—C14—H14B	109.5
C7—C8—H8A	109.5	H14A—C14—H14B	109.5
C7—C8—H8B	109.5	C13—C14—H14C	109.5
H8A—C8—H8B	109.5	H14A—C14—H14C	109.5
C7—C8—H8C	109.5	H14B—C14—H14C	109.5
H8A—C8—H8C	109.5		
			
C6—C1—C2—C3	1.0 (4)	C6—C1—C7—N1	−63.3 (3)
C7—C1—C2—C3	−179.6 (2)	C2—C1—C7—C8	−120.0 (3)
C1—C2—C3—C4	−1.1 (4)	C6—C1—C7—C8	59.4 (3)
C2—C3—C4—C5	0.5 (4)	O2—C9—C10—C11	−152.0 (2)
C3—C4—C5—C6	0.3 (4)	O1—C9—C10—C11	28.2 (3)
C2—C1—C6—C5	−0.3 (4)	C9—C10—C11—C12	−177.9 (2)
C7—C1—C6—C5	−179.7 (2)	C10—C11—C12—C13	−178.0 (2)
C4—C5—C6—C1	−0.4 (4)	C11—C12—C13—C14	−175.4 (3)
C2—C1—C7—N1	117.3 (2)		

### Hydrogen-bond geometry (Å, º)

**Table:**

*D*—H···*A*	*D*—H	H···*A*	*D*···*A*	*D*—H···*A*
N1—H1*A*···O2^i^	0.91	1.84	2.753 (3)	176
N1—H1*B*···O1	0.91	1.87	2.768 (3)	167
N1—H1*C*···O1^ii^	0.91	1.82	2.714 (2)	168

Symmetry codes: (i) *x*, *y*, *z*−1; (ii) −*x*+1, −*y*+2, *z*−1/2.
